# High-Throughput Label-free Single-Cell Proteomics Enabled by Multicolumn NanoLC with a 5-min Cycle Time

**DOI:** 10.21203/rs.3.rs-8042176/v1

**Published:** 2025-11-18

**Authors:** Chao Wang, Hsien-Jung L. Lin, Siqi Huang, Garrett D. Haynie, Kenneth S. Triggs, YenJou Chang, David V. Hansen, Thy Truong, Xiaofeng Xie, Ryan T. Kelly

**Affiliations:** a Department of Chemistry and Biochemistry, Brigham Young University, Provo, Utah 84602, United States;; b MicrOmics Technologies, LLC, Spanish Fork, Utah 84660, United States

## Abstract

Mass spectrometry (MS)-based single-cell proteomics (SCP) enables proteome-wide analysis at single-cell resolution, offering insights into cellular heterogeneity, biological processes, and disease mechanisms. However, conventional nanoLC-MS workflows are constrained by long gradients and low throughput, limiting their application to large cohorts. Here, we present a multicolumn nanoLC–MS platform that achieves 5-minute separation windows with 100% duty cycles at ~100 nL/min, enabling the analysis of up to 288 single cells per day with minimal additional hardware. The system provides stable peptide separation, negligible carryover, and robust retention-time reproducibility across 2,000 consecutive injections. Over the course of this study, we successfully analyzed more than 4,000 samples at nearly 288 samples per day (SPD) throughput. The platform identified ~4,400 proteins per 250 pg digest injection and an average of ~3,200 proteins per single HeLa cell with maxima exceeding 4,300, which is comparable to state-of-the-art longer-gradient workflows. Quantitative benchmarking with mixed-species standards confirmed accurate measurements across ~6,000 proteins. The workflow distinguished proteome profiles across hundreds of single cells, and profiling of RAW264.7 macrophages revealed LPS-induced markers and macrophage activation pathways. Together, these results establish a robust and scalable platform for high-throughput SCP, demonstrating the feasibility of thousands of single-cell analyses within a single study while maintaining deep proteome coverage and biological interpretability.

## Introduction

Quantifying cellular and tissue heterogeneity at the single-cell level across large populations offers critical insights into the protein-level diversity that are inaccessible through bulk measurements, ultimately enabling improved diagnostics and more effective therapeutic strategies^1–8^. Mass spectrometry-based approaches now enable profiling of protein expression at the single-cell level, and recent improvements in ion optics, detector speed, and acquisition strategies have substantially deepened coverage^9–23^. These developments have made it possible to reveal new aspects of cellular heterogeneity^24–28^.

Despite recent progress, throughput remains a critical bottleneck for SCP, as increasing analysis speed often comes at the expense of proteome coverage. To capture the full diversity of cellular states, thousands of single cells may need to be analyzed, and current LC-MS workflows (typically less than 100 SPD^29^) struggle with such large studies. Multiplexed strategies using isobaric^30–32^ or nonisobaric tags^33, 34^ have increased throughput by pooling labeled single cells, but ratio compression and isotopic interference often compromise quantitative accuracy^35, 36^, and proteome depth typically remains limited. The introduction of the TMTpro 35-plex set, for example, has enabled the analysis of up to 1,018 single cells per day, even with only ~30 LC runs per day (SPD)^37^. However, the number of reported proteins quantified per cell was lower than that of current label-free approaches, averaging 712 proteins per cell. Label-free SCP can reach greater proteome coverage, but at the cost of substantially lower throughput; conventional nanoLC-MS typically restrict analyses to only a few dozen single cells per day^29^.

One throughput limitation for label-free SCP lies in the inefficiency of conventional LC workflows, where significant portions of the analysis cycle are spent on overhead tasks such as sample pickup, column equilibration, gradient delay, and column cleanup that leave the mass spectrometer idle. Shortening gradients can reduce separation time, but overhead steps become proportionally more dominant, further lowering overall duty cycle. Increasing flow rates through microLC or capillaryLC setups shortens run times but sacrifices the sensitivity required for single-cell-level inputs^38–40^. Thus, a major challenge for the field is to achieve high throughput without compromising sensitivity.

Here, we present a multicolumn nanoLC-MS platform that achieves 5-minute separation cycles at ~100 nL/min flow rate, supporting throughput of up to 288 single cells per day. By multiplexing two analytical columns with dedicated storage loops^41, 42^, the system maintains a continuous 100% MS duty cycle while requiring only minimal hardware modifications.

We show that this design delivers negligible carryover, stable retention time stability across thousands of injections, and proteome coverage comparable to that of lower throughput analyses. Benchmarking against technical standards, mixed-species controls, and single-cell samples demonstrates that high throughput does not compromise sensitivity or reproducibility. Finally, we illustrate the biological utility of the platform by resolving proteomic differences between cell types and capturing inflammatory responses at single-cell resolution. Together, these results establish a practical and scalable solution to one of the most pressing challenges in SCP: enabling robust, high-throughput proteomic profiling at single-cell resolution. By bridging the gap between sensitivity and speed, the 5-minute multicolumn LC-MS system transforms SCP from a specialized method into a platform capable of addressing large-scale biological questions.

## Results

### Multicolumn LC-MS platform for high-throughput SCP

SCP has advanced rapidly in recent years, yet the throughput of LC-MS, particularly for label-free analyses, remains a limiting factor. In a conventional LC-MS setup, each sample undergoes sequential steps including sample pickup, column equilibration, loading onto a trap column, gradient elution from the trap to the analytical column, and column cleanup. Among these, only the gradient elution step produces mass spectra, and that may even include less informative spectra due to gradient delays. To address this inefficiency, we multiplexed these steps by using two analytical columns, and we avoided gradient delays through timing optimization and accelerating gradient generation via storage loops with minimal system complexity, adding only a single isocratic pump and one additional valve compared to standard systems.

Each sample is processed through the following parallelized steps: sample pickup, loading to the trap column, gradient elution from the trap column to a storage loop, and pushing from storage loop to analytical column for MS analysis (Supplementary Videos 1 and 2). Completing these processes within a 5-minute cycle requires precise coordination of multiple operations (Supplementary Video 3).

**Figure 1:**
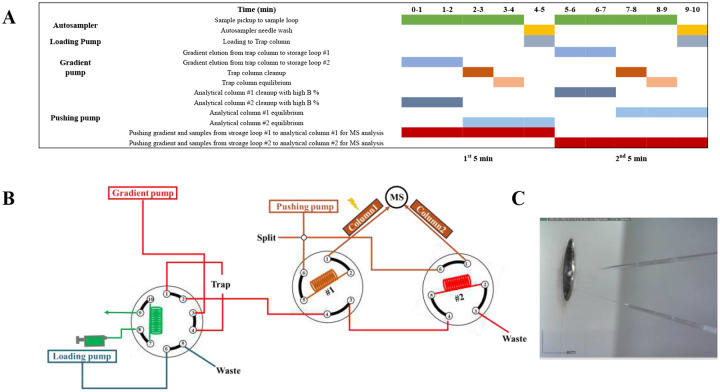
Two-column nanoLC system overview. **(A)** Minute-by-minute schematic diagrams. (**B)** Detailed connections of the multicolumn system. (**C)** Photomicrograph of two emitters positioned in front of the inlet of a FAIMS Pro interface.

The separate steps are illustrated in the timetable (Figure 1A), highlighting the individual stages of an LC-MS run with multiplexing implemented in two key areas. First, two analytical columns are operated alternately, each paired with a dedicated storage loop (as shown in the dual 6-port valve setup in Figure 1B). While one column elutes sample to the MS using the pre-formed gradient from storage loop #1, the next gradient is generated and loaded into storage loop #2, and vice versa. As illustrated in Figure 1C, the electrospray potential is applied to only one emitter at a time while the other is grounded, holding a droplet at its tip. This voltage control, managed by a simple adapter, ensures that regeneration or cleanup eluents from one column do not interfere with electrospray ionization on the other. Additional parallelization is applied to sample pickup, autosampler cleanup, trap column loading, and gradient elution to the storage loop (as illustrated in the 10-port valve in Figure 1B). Since sample pickup and gradient elution share the same valve position, they can occur simultaneously. Likewise, autosampler cleanup and sample loading occur in parallel. In this case, during the first 4 minutes of each cycle, while the eluent of the previous sample transfers from trap column to storage loop, the autosampler picks up the next sample. After valve switching, the autosampler is cleaned while the newly drawn sample is loaded onto the trap column.

With these combined strategies, we achieve true 5-minute analysis cycles with a 5-minute separation window and maintain a near-100% MS duty cycle at a low flow rate of ~100 nL/min. One minor exception is that there is a brief wait period between two injections caused by the downloading process of the MS acquisition software. This may be remedied by collecting data for a number of samples in the same raw file and then splitting the file after collection. As shown in Table 1, we can obtain a chromatogram every 5 min after the 3^rd^ 5 min segment once the operation of the system is fully parallelized. This configuration enables high-throughput analysis of up to 288 samples per day (SPD) for SCP or other low-input analyses. In contrast to alternative high-throughput solutions such as Evosep, whose Whisper Zoom methods enable 20–120 SPD at higher flow rates, reduced sensitivity and lower duty cycles, the present system preserves ultra-low-flow operation that benefits single-cell proteomics. The ability to simultaneously achieve high throughput and high sensitivity positions this platform in a distinct performance regime within SCP and proteomics more broadly.

### Separation performance characteristics and long-term robustness

**Figure 2:**
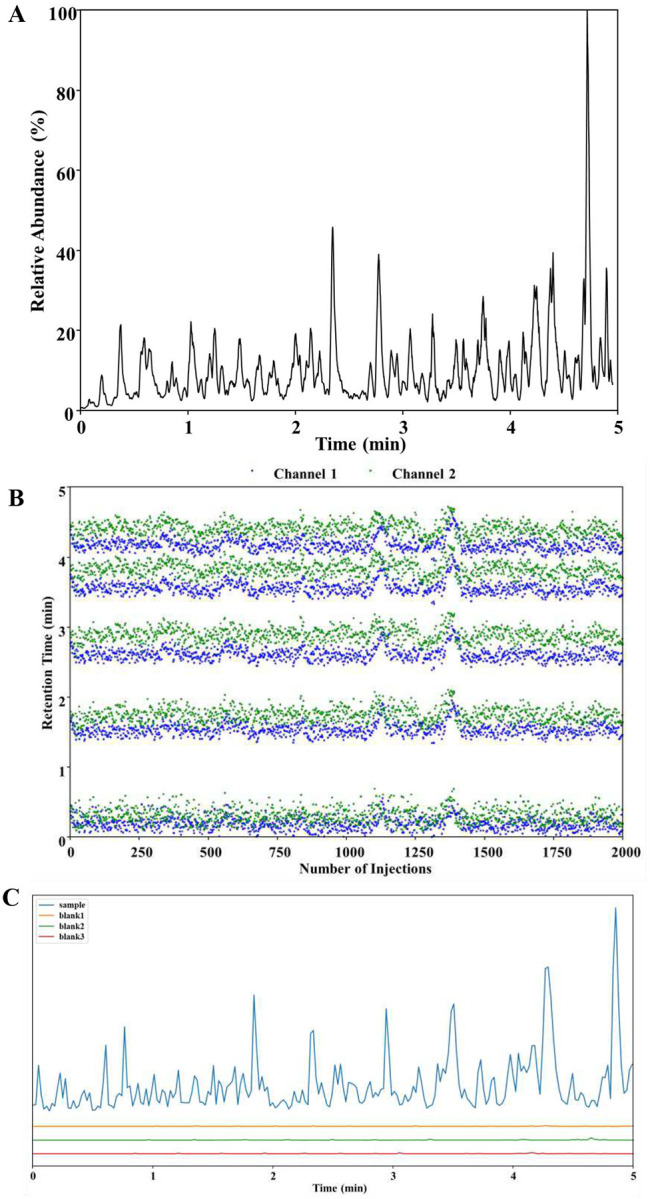
Separation performance evaluation of the multicolumn LC system. **(A)** Base peak chromatogram of 2 ng HeLa digests with a 5 min cycle time. **(B)** Retention time reproducibility for 5 peptides over 2,000 continuous analyses. For each of the 5 peptides, elution times from one column are shown in green and those from the other column are shown in blue. Retention time changes during the analysis are due to temperature fluctuations in the lab space. **(C)** Base peak chromatograms of 2 ng HeLa digests runs followed by three blank runs, indicating negligible column carryover.

For a 5-minute separation with ~100% duty cycle, it is essential to ensure that the actual separation window fits entirely within this time frame. The separation window of the multicolumn system was evaluated using 2 ng human protein digest (equivalent to ~10 cells). By adjusting the accelerated gradient and the timing of the 6-port valve switch, we can ensure that the separation window is well aligned with the 5-minute MS acquisition time. That is, the separation window is neither too narrow, which would underutilize the 5-minute acquisition time, nor too wide to extend beyond it, which could result in sample elution loss. As shown in Figure 2A, the peptide elution window is well distributed across the 5-minute MS acquisition time, enabling the high throughput of ~288 samples per day with an actual 5-minute analysis time per sample.

We next assessed retention time stability over extended operation, a common concern in high-throughput analyses. To evaluate retention time (RT) stability, we analyzed 2,000 samples without interruption, extracting the retention times for five peptides that spanned the separation window across all runs. As shown in Figure 2B, for a single analytical column, the standard deviation of retention time ranged from 0.10 min (6.0 s) to 0.16 min (9.6 s). Some of this was due to temperature fluctuations in the room, as the columns were unheated. When the two channels were combined, the RT distributions became slightly broader due to the inherent challenge in obtaining two identical columns. Nevertheless, the combined RT standard deviation only broadened slightly to 8.4 to 10.2 s. This demonstrates that the system offers excellent RT stability and high robustness, making it capable of continuously analyzing thousands of samples.

Sample carryover between injections is a critical concern in proteomics, as it can lead to false positives and quantitative inaccuracy. To address this concern, we evaluated the carryover of the system using 2 ng HeLa tryptic digests. As shown in Figure 2B, no peptide peaks were observed in the blank runs following the sample injection, indicating negligible carryover. The intensity for blank runs was less than 1% compared to the sample runs (Table S1). This low carryover is attributed to the rigorous washing procedure after the accelerated gradient for trap column cleanup (Figure S2) and the use of 80% ACN in the isocratic ‘pushing pump’ for analytical column cleanup. The low carryover for the system ensures minimal cross-contamination, making it suitable for high-throughput analysis of large cohorts of samples.

### Column-to-column consistency supports pooled analysis

Since all experiments in this study were analyzed across two analytical columns, spanning multiple plates and acquisition days, it was essential to verify that technical variability did not mask or distort biologically meaningful differences. As a first step, we assessed whether protein identifications and intensity-based variation were comparable between the two columns, and whether results could be fairly combined for downstream analysis. To this end, we analyzed 376 technical replicate injections of a 250 pg HeLa digest standard, a commonly accepted proxy for the approximate protein content of a single mammalian cell. There were 188 injections on each analytical column, distributed across two sample plates and interspersed with other samples such that these samples were acquired over the course of seven days. The 250 pg HeLa digest standard was prepared at a concentration of 250 pg/μL, and 1 μL was injected per run.

**Figure 3:**
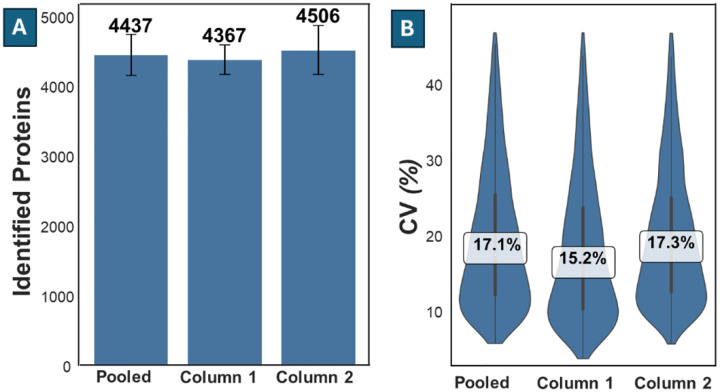
Protein identification and quantification reproducibility across two analytical columns. **(A)** Average number of protein identifications from 376 injections of 250 pg HeLa digest standard, shown for pooled data (all runs) and for runs acquired on Column 1 and Column 2 separately. Error bars indicate standard deviation. **(B)** Distribution of coefficients of variation (CV) for protein intensities across replicates. Median CV values are indicated.

The pooled dataset yielded an average coverage of 4438 ± 295 protein groups, with a maximum of 4769 protein groups. Separately, column 1 and column 2 produced averages of 4367 ± 208 and 4506 ± 349, respectively, corresponding to a difference of just 3.13% (Figure 3A).

In terms of quantification reproducibility, the median coefficient of variation (CV) of protein intensity was 17.1% across all runs. Column 1 and column 2 showed median CVs of 15.2% and 17.3%, respectively (Figure 3B). These observed CVs are in line with other measurements. For example in our ‘$ 0 proteome’ study^43^, which employed an 11.4-min gradient, we observed median CV values below 10% for sample inputs greater than 1 ng, and between 10% and 20% for inputs of 1 ng or below. In the same study, we observed minimal change in CV for Orbitrap Astral-acquired data as the gradient decreased from 40 min to 5 min. As such, the run-to-run variability is similar to that observed in other low-input studies, and the system performance is sufficiently stable to justify pooling MS runs from both columns for subsequent analysis without introducing column-specific bias.

### Proteome Coverage and Quantification Across Single-Cell Sampling Formats

**Figure 4.**
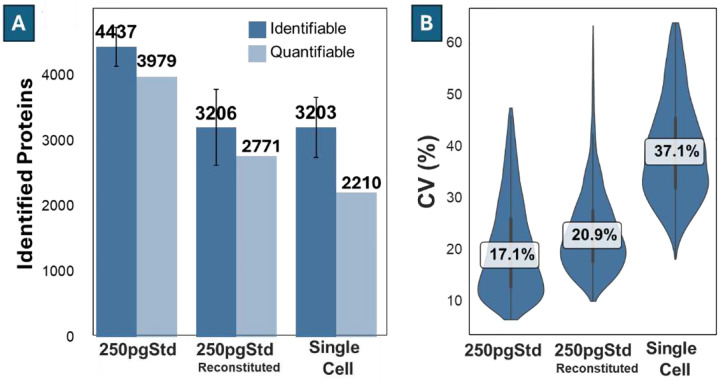
Protein identification and quantification reproducibility across two analytical from single cell-level Input. **(A)** Average number of identifiable proteins identified from 250 pg HeLa digest standard (1 μL injection), reconstituted 250 pg standard, and isolated single cells. Error bars show standard deviation. The quantifiable column indicates the number of proteins retained across replicates after missing value and IQR filtering. **(B)** Distribution of protein intensity CV across replicates.

Next, we assessed proteome coverage and run-to-run quantification variability at the single-cell level using both HeLa digest standards and isolated HeLa single cells, analyzed with different injection methods. In addition to the 1 μL injection of a 250 pg/μL HeLa digest standard described in the above section, we also evaluated a reconstituted injection method. In this method, 0.20 μL of a .25 ng/μL HeLa digest standard was dispensed into a 384-well PCR plate, allowed to dry, then reconstituted to a larger volume by the autosampler before being aspirated into the sample loop. Across 228 runs using this reconstituted format, we identified an average of 3206 ± 58 proteins per run, with a maximum of 4159 proteins. This reconstituted 250 pg HeLa digest standard was used as a reference to assess the technical differences introduced by sampling methods, as it shares the same sampling procedure used for isolated single cells. For isolated HeLa single cells, we identified an average of 3203 ± 460 proteins across 68 runs, with a maximum of 4312 proteins (Figure 4A). The relative difference in proteome coverage bet ween the μL injection method (no reconstitution needed) and the reconstituted 250 pg standard was 32.2, while the difference between the reconstituted standard and isolated single cells was only 0.1%. This suggests that the primary source of losses at the single-cell level arises from incomplete recovery of sample following drying. Given this observation, it may be beneficial to prepare single-cell samples in somewhat larger volumes (e.g., ≥1 μL) to avoid needing to reconstitute dried samples, even if the larger-volume preparation is slightly less efficient. This will be investigated in greater detail in a future study.

We filtered the data by excluding proteins having >33% missing values across replicates, and we applied a 1.5× Interquartile Range (IQR) filter on CV of protein intensity to remove outliers. After filtering, the number of quantifiable proteins for the 250 pg HeLa digest standard, the reconstituted 250 pg HeLa digest standard, and isolated HeLa single cells were 3979, 2771, and 2210, respectively. The corresponding ratios of quantifiable to identified proteins were 90%, 86%, and 69% (Figure 4A). Among these quantifiable proteins, the median CV of protein intensities was 7. for the 250 pg digest standard, 20.9 for the reconstituted standard, and 37. for the isolated single cells (Figure 4B). The slightly elevated CVs observed in reconstituted standard compared to the 1 μL injection standard indicates the quantification variability introduced by the sampling technique. The further increase in CV for isolated single cells compared to reconstituted standards reflects the quantification variability introduced due to biological heterogeneity in addition to the technical variation.

To place these results in context, we compared them with two studies published in 2025 that also highlighted the high sensitivity of single-cell proteomics using the Orbitrap Astral mass spectrometer. Those studies reported an average of 5,102 proteins from 250 pg of HeLa digest standard with an 80 SPD method, 4,879 and 3,166 proteins from A549 and H460 single cells with a 50 SPD method^11^, and 4,679 proteins from HeLa single cells with a 120 SPD method^10^. Our results demonstrate only modestly reduced proteome coverage while enabling >2–5× greater throughput.

These results also highlight the sensitivity and robustness of the 5-minute LC-MS system in achieving deep proteome coverage and consistent quantification at the single-cell input level. The observed decrease in performance for single-cell samples is largely attributed to sampling technique rather than input material. While further optimization on sampling single cell sample may enhance both proteome coverage and quantification reproducibility in single-cell analyses, the results presented here validate the system’s sensitivity, reproducibility, and robustness for both standard and single-cell inputs, establishing confidence in its suitability for single-cell type biological studies.

### Quantitative Performance with mixed-species standards

To further assess the quantitative performance of our 5-minute multicolumn LC system, we used standards that combined human, yeast, and *E. coli* (HYE) peptides mixed at defined ratios as originally described by Navarro et al^44, 45^. The HYE standard is widely used for benchmarking quantification accuracy in proteomics because it provides a ground-truth comparison of relative abundances as the three distinct proteomes are combined at known proportions. We used the HYE 124 mixtures: HYE A (H:Y:E = 65:30:5) and HYE B (H:Y:E = 65:15:20). The amount of human peptide was 250 pg in both the A and B mixtures.

**Figure 5:**
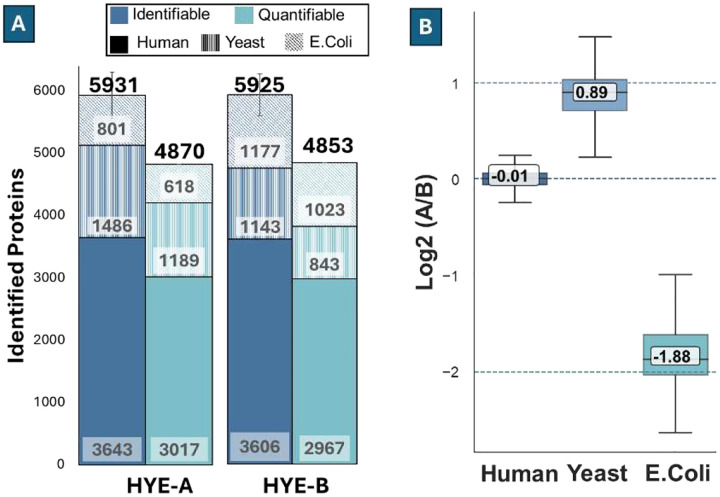
Quantification accuracy and reproducibility using HYE samples. **(A)** Average numbers of identified proteins and quantifiable proteins after filtering. For HYE-A and HYE-B mixtures, the total numbers are shown above each column, while the contributions from human, yeast, and *E. coli* (bottom to top) are labeled inside the stacked bars. **(B)** Box plot of the measured log_2_ ratios of human, yeast, and *E. coli* proteins, with median values labeled.

Combining all species, identified 5931±368 proteins, with a maximum of 6536 across 330 replicates in HYE A; and 5925 ± 339 proteins, with a maximum of 6155 across 336 replicates in HYE B. The number of quantifiable proteins after applying a 33% missing value filter and 1.5× IQR outlier removal is 4870 and 4853, respectively, for HYE A and HYE B, such that ~82% of the identifiable proteins were retained in both mixtures (Figure 5A). Although the human input was held constant at 250 pg, there were fewer human proteins identified in the HYE mixtures (HYE A: 3643 ± 213 protein groups; HYE B: 3606 ± 204 protein groups) than in human-only runs (4438 ± 295). This is a well-known matrix effect^46–48^ in mixed proteomes: additional yeast/*E. coli* peptides increase coelution, ion-suppression and spectral complexity in addition to cross-species peptide homology that tightens FDR filtering. Together, these factors reduce sampling depth for the human subset with the 5-minute gradient. In terms of quantification reproducibility, the median CV of protein intensity across replicates was 22.3% for HYE A and 22.9% for HYE B. The higher CV compared to HeLa-only runs likely reflects the same matrix effects described above.

To assess quantitative accuracy, we compared ratios of the human, yeast, and E. coli components in the HYE standard against their kno wn log2 ratios (H:Y:E= :0:−2). The measured ratios were 0.89, −0.0, and − .88, yielding absolute errors of 0., 0.0, and 0.12, respectively, for human, yeast, and E. coli (Figure 5B), and corresponding relative errors of 11%, 1%, and 6%. These results are well within the typical range of quantitative accuracy reported for label-free DIA analysis.^49, 50^ The close agreement between measured and expected values confirms that the 5-minute LC-MS method maintains high quantitative capability even in complex, mixed-proteome backgrounds.

### Differentiating cell types and treatment groups

**Figure 6:**
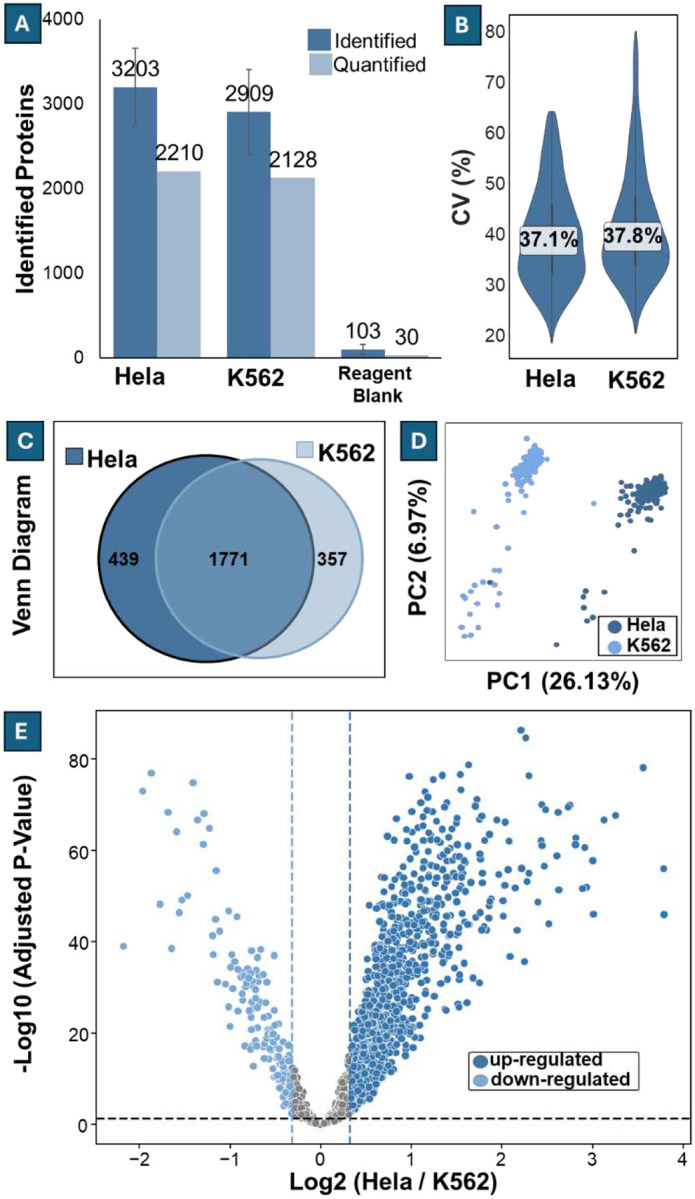
Differential Expression of Hela and K562 Single cells. **(A)** Average numbers of identified and quantifiable proteins per cell for HeLa (n = 168) and K562 (n = 190). Error bars indicate standard deviation. **(B)** Distribution of protein intensity CVs across quantifiable proteins, with median CVs labeled. **(C)** Venn diagram showing overlap of quantifiable proteins, with 1771 shared, 439 unique to HeLa, and 357 unique to K562. **(D)** Principal component analysis (PCA) of protein intensities reveals a clear separation of HeLa and K562 single cells. **(E)** Volcano plot of differential protein expression between HeLa and K562 cells, highlighting significantly up-(n=976) and downregulated (n=162) proteins (log_2_ fold change ≥ 0.32, adjusted p < 0.05).

Having demonstrated that the 5-minute LC-MS system can quantify differences using standard samples (e.g., HeLa digest and HYE), we next assessed its ability to detect biologically relevant variation across distinct cell types and within the same cell type under different treatments. We began by comparing two widely used human cell lines: HeLa and K562. In total, we analyzed 168 HeLa cells and 190 K562 cells. HeLa cells yielded an average of 3203 ± 460 identified proteins per cell, while K562 cells averaged 2909 ± 502. After filtering proteins for ≤33 missing values and removing outliers using a 1.5× IQR filter, 2210 and 2128 proteins remained quantifiable in HeLa and K562 cells, respectively, such that ~71% were retained in both groups (Figure 6A). Protein intensity reproducibility, assessed by the median CV, was 37.1% for HeLa and 37.8% for K562 (Figure 6B). Despite the similarity in quantifiable coverage and variability, clear differences were observed between the two cell types. Of the quantified proteins, 1771 were shared, while 439 and 357 were unique to HeLa and K562, respectively (Figure 6C). Principal component analysis (PCA) revealed distinct clustering patterns by cell type (Figure 6D). Differential expression analysis using Welch’s t-test identified 38 significantly different proteins (log_2_ fold change ≥ 0.32, adjusted p < 0.05), including 976 upregulated and 62 do wnregulated in HeLa compared to K562 (Figure 6E). These results demonstrate that our 5-minute multicolumn LC system provides sufficient proteomic depth, reproducibility, and sensitivity to resolve substantial biological differences between single cells of distinct origin.

**Figure 7:**
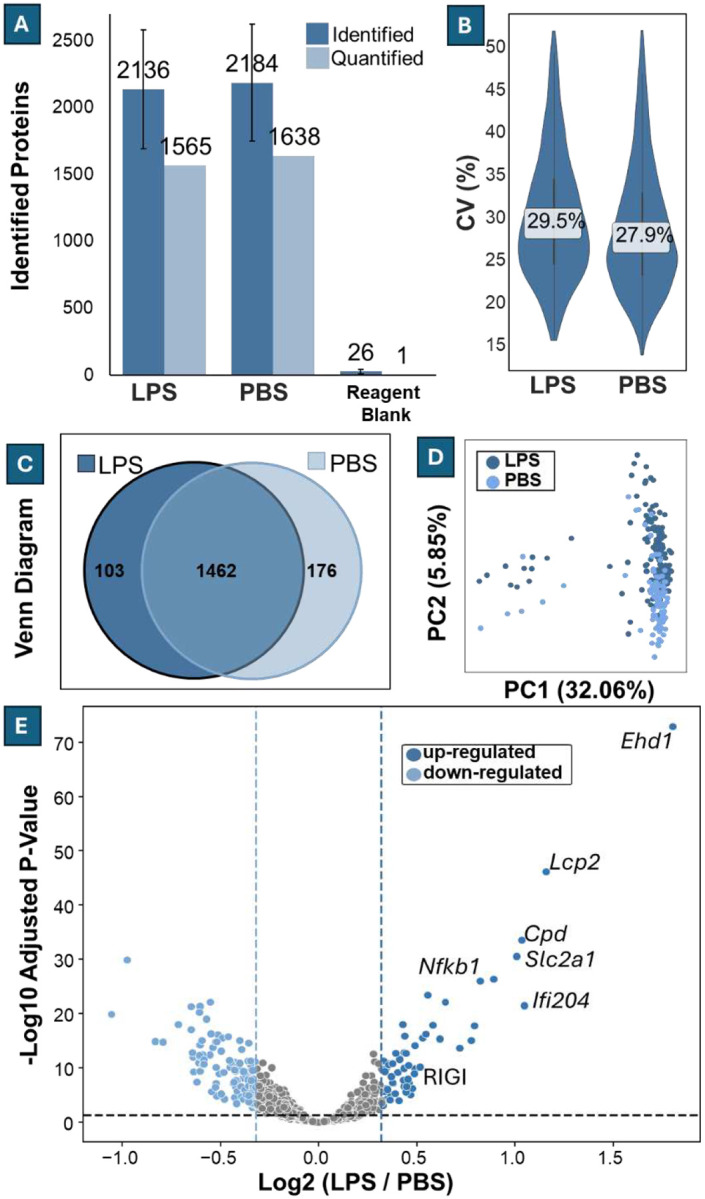
Differential Expression of RAW264.7 Cells infected with LPS. **(A)** Average numbers of identified and quantifiable proteins per cell in LPS-treated (n = 170) and PBS control (n = 95) groups. Error bars represent standard deviation. **(B)** Distribution of protein intensity CVs across quantifiable proteins, with median CVs labeled. **(C)** Venn diagram showing overlap of quantifiable proteins between conditions, with 1462 shared, 103 unique to LPS, and 176 unique to PBS. **(D)** Principal component analysis (PCA) of protein intensities, showing partial separation between LPS and PBS single cells. **(E)** Volcano plot of differential protein expression between LPS and PBS groups, highlighting 73 significantly regulated proteins (log_2_ fold change ≥ 0.32, adjusted p < 0.05), known LPS-induced markers such as Nfkb1, Ifi204, Lcp2, and Ehd1are labeled.

We next evaluated the performance in detecting treatment-induced changes within a single cell type under inflammatory stimulation, a more challenging benchmark than cell-type separation. For this, mouse macrophage RAW264.7 cells were treated with 40 ng/mL lipopolysaccharide (LPS) for 16–18 hours, while control cells received an equal volume of PBS. LPS stimulation activates innate immune signaling, particularly the NF-κB path way, leading to a broad but moderate proteomic response^51–58^. In total, we analyzed 170 LPS-treated and 95 PBS-treated single cells (control). The LPS group yielded an average of 2 36 ± 444 identified proteins, with 565 retained after filtering. The PBS group yielded 2 84 ± 437 identified and 638 quantified proteins (Figure 7A). Approximately 74% of identified proteins were quantifiable, which is consistent with ratios observed in our HeLa and K562 single-cell datasets. The median coefficients of variation (CVs) for protein intensity were more modest at 29.5% (LPS) and 27.9% (PBS) (Figure 7B). Of the quantified proteins, 1462 were shared between the two groups, with 103 unique to LPS and 176 unique to PBS (Figure 7C). Principal component analysis (PCA) showed partial overlap between LPS and PBS clusters (Figure 7D). A Welch’s t-test identified 173 differentially expressed proteins (fold change ≥ .25, adjusted *p* < 0.05), including 56 upregulated and 7 do wnregulated proteins. The upregulated proteins were predominantly inflammation-related, including well-known LPS-induced targets such as *Nfkb1*, *Ehd1*, and *Ifi204* (Figure 7E)^51–58^.

Compared to a 2022 study that used a 60-minute gradient to analyze LPS-stimulated RAW264.7 cells^59^, our system demonstrated substantial improvements. That study identified a median of 451 proteins across 155 cells, whereas our method identified a median of 2158 proteins across 265 cells—a nearly 5-fold increase using only 8% of the LC gradient time. While the 2022 study retained approximately 31% of identified proteins after filtering, our pipeline retained 74%, even with stricter missing value and outlier exclusion criteria. The earlier study reported 250 significant proteins across three conditions, whereas we observed 173 significant proteins from just t wo conditions. This difference may be due to the lower LPS dose (40 ng/mL vs. 00 ng/μL) and shorter treatment duration (16–18 hours vs. 24–48 hours) in our experiment. Nonetheless, we still detected clear inflammatory responses, including key LPS-induced markers.

**Figure 8:**
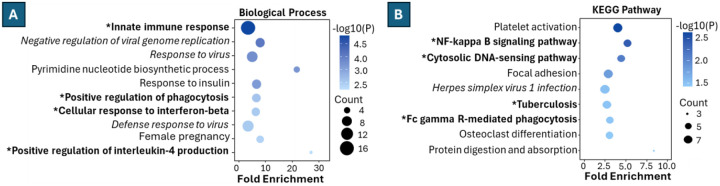
Representative Significant Gene Ontology Terms. **(A)** Biological Process, BP terms significantly enriched among differentially expressed proteins. The x-axis represents enrichment ratio, bubble size reflects the number of proteins in each category, and bubble color indicates −log10(p-value). **(B)** KEGG pathway enrichment analysis of differentially expressed proteins, with visualization parameters as in panel A. * & Bolded indicates terms that are directly related to LPS stimulation, where Italic terms are related.

We next subjected the 56 upregulated proteins and 103 unique to LPS treatment to gene ontology analysis using DAVID^60, 61^. Among 33 significant Biological Process categories (p < 0.05), 11 corresponded to canonical LPS biology, including innate immune response, macrophage activation, regulation of phagocytosis, interferon signaling, regulation of IL-4/6, tumor necrosis factor production, and response to oxidative stress. An additional four viral-annotated terms (e.g., *antiviral innate immune response*) reflect shared innate immune pathways activated by both viral and bacterial stimuli (Figure 8A). KEGG pathway analysis identified 9 significant pathways, including 4 directly relevant to LPS responses: *NF-κB signaling, Cytosolic DNA-sensing, Tuberculosis*, and *FcγR-mediated phagocytosis* (Figure 8B)^62–67^. Together, these enrichment results show that hallmark macrophage activation pathways were consistently captured, even when using a 5-minute gradient.

It is also worth noting that the data from the previous study^59^ were acquired on an Orbitrap Lumos mass spectrometer, while our data were generated using the Orbitrap Astral mass spectrometer. Since the introduction of Astral, overall protein identification rates in single-cell proteomics have improved substantially. This comparison not only highlights the evolution of the field but also demonstrates that our 5-minute LC-MS system can sensitively and reproducibly quantify subtle biological changes at single-cell resolution, challenging the paradigm of previous generation mass spectrometers for which longer gradients were necessary for high-quality proteomic data. Indeed, a recent study in our group showed minimal added proteome depth or reproducibility when increasing the gradient length from 5 min. to up to 60 min^43^ using either the Orbitrap Astral or timsTOF Ultra 2 mass spectrometer. Together, the K562–HeLa comparison and LPS stimulation experiments illustrate the sensitivity of our 5-minute multicolumn LC system across diverse biological contexts. Whether resolving distinct cellular identities or detecting subtle treatment-induced changes within the same cell type, the system consistently delivers deep proteome coverage and reproducible quantification at the single-cell level.

## Discussion

This study establishes a multicolumn LC-MS system for high-throughput SCP, overcoming a fundamental bottleneck in current SCP workflows. By implementing the LC system with 5-minute duty cycle, we demonstrate that high-throughput SCP profiling is achievable for up to 288 single cells per day with minimal compromise in sensitivity or reproducibility. This throughput provides a substantial improvement over prior workflows, overcoming the long analysis times that have historically limited the scale of SCP studies.

Qualitative and quantitative assessments across technical standards, mixed-species benchmarks, and single-cell samples show that the platform achieves deep proteome coverage, up to 4,300 proteins per single HeLa cell, while maintaining stringent retention-time stability, minimal carryover, and reproducibility across thousands of samples. Compared with recent Astral-based workflows using 40–120 SPD methods, our system delivers only slightly reduced proteome depth at more than double the throughput, reshaping expectations for the balance between sensitivity and speed in single-cell proteomics.

The platform also enables resolution of biologically meaningful variation at the single-cell level. Comparative analysis of HeLa and K562 cells identified over 1,100 significantly different proteins, while profiling RAW264.7 macrophages revealed LPS-induced markers and macrophage activation pathways. Notably, the number of proteins quantified in single cells exceeded those reported in prior 60-minute studies of the same system by nearly fivefold, underscoring that short-gradient methods (combined with latest-generation MS) can both accelerate and enhance biological discovery.

Together, these advances directly address two of the most pressing needs in single-cell proteomics: throughput and robustness. The ability to analyze 288 single cells per day with minimal carryover and stable retention times enables large-scale studies of cellular heterogeneity, perturbation responses, and patient cohorts that were previously impractical. Because the system requires minimal hardware modifications (an additional pump and valve assembly) it can be readily adopted on existing low-flow LC setups, lowering the barrier for broad implementation.

In summary, the high-throughput multicolumn LC system is a valuable contribution to evolve the throughput of SCP to the next level, helping to transition the field of SCP from method development to the biological toolbox. By enabling routine analysis of 288 single cells per day, this method transforms the scale at which biological and clinical questions can be addressed, opening the door to proteome-wide investigations in single-cell atlases and translational studies. To date, we have analyzed more than 4,000 samples using this platform, and with continued advances in mass spectrometer performance, throughput of 500–1,000 cells per day at comparable depth appears to be within reach.

## Methods

### Sample Preparations

#### Cell Culture and treatment:

HeLa cells were cultured in 10-cm tissue culture plates and K562 cells in 75 mL culture flasks using 10 mL of either 1x DMEM (Corning, 10–013-CV) or 1x RPMI-1640 Medium (Cytiva, SH30027.01), respectively. Both media were supplemented, making complete media, with 10% fetal bovine serum (FBS) and 1% penicillin-streptomycin. All plates were incubated at 37 °C in a humidified incubator with 5 CO_2_. Once HeLa cells reached confluency, they were rinsed with 5 mL of 1× phosphate-buffered saline (PBS), detached using 4 mL of 0.25% trypsin-EDTA (Gibco, 25200–072), and incubated at 37°C for 4 minutes. Trypsin activity was quenched by the addition of 10 mL of complete medium. K562 cells, which grow in suspension, were gently scraped from the flask using a cell scraper (Sarstedt, Ref# 83.3951) and collected once confluency was reached.

RAW264.7 cells were cultured in 10-cm plates using complete Corning DMEM supplemented with 10% fetal bovine serum (FBS), 1% penicillin-streptomycin and 2 mM Gibco GlutaMAX. When cells reached approximately 60–70% confluency, they were washed once with PBS and fresh complete medium was added. Lipopolysaccharide (LPS; *Escherichia coli* O111:B4, Sigma-Aldrich, L5293) was added to achieve a final concentration of 40 ng/mL by adding 400 μL of a 1 μg/mL LPS stock solution. For control samples, an equivalent volume (400 μL) of PBS was added instead of LPS. All plates were incubated at 37°C in a humidified incubator with 5 CO_2_ for 6–18 hours, then collected following the procedure described for HeLa cells above.

#### Cell Wash:

All cell types were pelleted separately by centrifugation at 200 ×g for 10 minutes, resuspended in 2 mL of 1× PBS, and passed through a 40 μm filter. The cells were washed further by subsequent repelleting at 200 ×g for 5 min., resuspending in 1 mL of 1× PBS and repeating these steps to remove residual media and enzymes. Cell viability and diameter were assessed using Trypan Blue Stain (Gibco, Ref# 15250–061) and the Logos Luna II Automated Cell Counter. HeLa cells exhibited an average viability of 88.7%, while K562 cells showed a viability of 92.3%. The RAW264.7 cells exhibited a viability of 82.20% for the control group, and a viability of 83.55% for the lipopolysaccharide-treated group. All cell types were adjusted to a final concentration of 2 × 0^5^ cells/mL before isolation.

#### Single-Cell Isolation:

The single-cell sample preparation followed the protocol published previously^68–70^. In brief, single-cell dispensing was performed into DDM-treated 384-well PCR plates using the Tecan UNO dispenser (Männedorf, Switzerland). During dispensing, size-based gating was applied with a diameter range of 15–17 μm for HeLa cells and 12–14 μm for both K562 and RAW264.7 cells. Plates were centrifuged at 380 ×g immediately following dispensing to ensure cells were driven to the bottom of each well. HeLa and K562 cells were dispensed to the same plate. Each plate contained 100 HeLa cells and 100 K562 cells, and a total of two plates were analyzed by LC/MS. As for RAW 264.7 cells, 100 cells for each control and lipopolysaccharide treated group were sorted to the same plate.

#### Digestion:

One-pot workflow was exclusively applied in single-cell proteomics sample preparation to avoid sample loss during multiple transfer steps^68–72^. Immediately after isolation of all cell types, wells containing single cells were subjected to in-well proteolytic digestion^70^. A 200 nL volume of digestion solution was dispensed into each well using the Tecan UNO dispenser. The digestion mix consisted of 10 μL of 0.2 ng/nL Rapid Trypsin/Lys-C (Promega, Cat# V507C) in Resuspension Buffer (Promega, Cat# V181A), 10 μL of 1% DDM in Rapid Digest Buffer (Promega, Cat# VA106A), and 180 μL of Rapid Digest Buffer, resulting in final concentrations of 10 ng/μL Trypsin/Lys-C and 0.05% DDM. This corresponds to an enzyme-to-substrate (E:S) ratio of 10:1, assuming ~200 pg of total protein per cell. The digest mix solution was also dispensed into empty wells for the purpose of reagent blanks. Plates were centrifuged, sealed with a silicone mat, wrapped in aluminum foil, and incubated in a humidified oven at 70°C for 1 hour. After digestion, plates were cooled to 4 °C for 15 min., sealed with aluminum sealing foil, and stored at −20°C until LC-MS/MS analysis.

**HeLa Digest standard** was prepared using Pierce^™^ HeLa Protein Digest Standard (ThermoFisher, Ref# 88329). Dilutions were prepared at 10 ng/μL and 5 ng/μL for library creation, and at 250 pg/μL with 0.1% formic acid for system evaluation. The HeLa Digest standard was also prepared at 1.25 ng/μL and dispensed with the D100 HP dispenser at 200 nL per well to DDM-treated 384-well plates for the evaluation of sample recovery following reconstitution.

**K562 Digest Standard** was prepared from Promega MS-Compatible Human Digest (Promega, V6951) and diluted to 10 ng/uL with 0.1% Formic Acid to 10n/uL for library creation.

#### HYE Digest Standard:

The HYE mixture (Human, Yeast, and *E. coli* peptides) was prepared using Pierce^™^ HeLa Protein Digest Standard (ThermoFisher, Ref# 88329), Yeast protein extract (Promega, Cat# V7461), and MassPREP^™^ *E. coli* Digest Standard (Waters, Part#186003196). Two mixtures were prepared: HYE A and HYE B. For HYE A, 100 μL each of the three components—HeLa, Yeast, and E. coli digests were combined, yielding final concentrations of 20 ng/μL, 9.23 ng/μL, and 1.54 ng/μL, respectively. HYE B was prepared similarly, with final concentrations of 20 ng/μL, 4.62 ng/μL, and 6.15 ng/μL, respectively. The solution was diluted to achieve a final HeLa component concentration of 10 ng/μL for library creation and 250 pg/μL for system evaluation.

#### DDM Plate Treatment:

384-well PCR plates (Eppendorf, Cat# 951020702) were treated with 0.01% *n*-dodecyl β-D-maltoside (DDM; Sigma-Aldrich, Cat# D4641–1G) in Milli-Q water by dispensing 20 μL into each well using a multichannel micropipette. Plates were centrifuged to ensure DDM coated the well bottoms and were incubated at room temperature for 30 min. The DDM solution was then removed by flicking, followed by inverting the plates and centrifuging at 380 × *g* to remove residual liquid.

### Multicolumn nanoLC setup

Based on the different multicolumn setup published before^73–76^, the multicolumn LC system was constructed as described previously^76^. The trap columns (00 μm i.d. × 35 mm long and packed with 3.5 μm diameter media having 100 Å pores), analytical columns (30 μm i.d. × 50 mm long; 1.7 μm diameter, 100 Å C18 particles) and emitters (0 μm i.d.) were purchased from MicrOmics Technologies, LLC (Spanish Fork, UT). Mobile phase A and B were LC-MS-grade water with 0.1% formic acid and acetonitrile with 0.1 % formic acid (Honeywell, Charlotte, NC). The minute-by-minute multicolumn nanoLC system setup is shown in Figure S1. Samples were first aspirated into the sample loop (a and b) followed by loading onto the trap column at 5 μL/min for ~1 min (c), and then samples were eluted to the storage loop from the trap column (d). The eluent, including the sample, gradient, and the column equilibration solvent A, were stored inside the storage loop. After switching the 6-port valve, an additional isocratic pushing pump containing 0.1 % formic acid in 80% ACN served to push the eluents of the storage loop to the analytical column at the flow rate of ~100 nL/min (d and e). After that, the pushing pump was directly connected to the analytical column for cleanup (f).

### Mass Spectrometry

The retention time stability evaluation was performed in LTQ Linear Ion Trap mass spectrometer (Thermo Fisher Scientific, San Jose, CA). The ion transfer tube temperature was set as 250 °C and the spray voltage was set as 1.8 kv. Full scan was applied at positive mode with m/z ranging from 375–975.

The evaluation of carryover was performed using Orbitrap Exploris 480 mass spectrometer (Thermo Fisher Scientific). The ion transfer tube temperature was set as 250 °C and the spray voltage was set as 2.1 kV. Full scan was applied in positive ionization mode with m/z ranging from 375–975 at 120K resolution. Injection time was set to 22 ms and AGC target was set to 300%. The RF lens was set as 50%. MS/MS scan mode was DIA with isolation window set to 16 Th for m/z range of 400–800 to generate 25 scan events. HCD collision energy was 30% with AGC set at 2000% and injection time set to auto.

The analysis of 250 pg HeLa digests, HYE and all single-cell samples were performed on the Orbitrap Astral mass spectrometer (Thermo Fisher Scientific, San Jose, CA). FAIMS was applied with the carrier gas flow set at 3.5 L/min and CV set at −45 V. The ion transfer tube temperature was set as 250 °C. Full scan was applied with m/z ranging from 400–800 at 240K resolution. Injection time was 100 ms and AGC target was 500%. RF lens was 45%. MS/MS scan range was set as 150–2000 with DIA isolation window set to 8 Th, generate 49 scan events. Normalized HCD collision energy was 25% with AGC set to 800% and maximum injection time set to 12 ms.

### Data analysis and bioinformatics

All MS raw files were identified and quantified using DIA-NN 1.9 with Human, Mouse, Yeast, and Ecoli UniProt protein database downloaded in December 2024 through MSConnect^77^ at near-real-time to monitor system stability. The data reported in this study were processed using Spectronaut (v19.8) directDIA+ workflow, using the same UniProt protein database that was used for MSConnect. Trypsin/Lys-C was specified as the digestion enzyme, with a peptide length range of 7–52 amino acids and up to two missed cleavages. A maximum of five modifications were allowed per peptide, including fixed carbamidomethylation and variable modifications such as protein N-terminal acetylation and methionine oxidation. The false discovery rate for PSMs, peptides, and protein groups was controlled at 1%. Quantification was performed at the MS1 level, and cross-run normalization was disabled.

Five Spectronaut projects were created to organize and analyze the datasets. The HeLa digest standard project included 250 pg, 5 ng, and 10 ng inputs injected directly, while a second project analyzed 250 pg HeLa digests prepared by the reconstituted injection mode. The third project contained HYE Mix A and B at 250 pg, 5 ng, and 10 ng input levels to benchmark quantification accuracy across known organismal ratios. The fourth project included single-cell HeLa and K562 analyses, reagent blanks, and 10 ng digests from each cell type for reference. The final project analyzed RAW 264.7 macrophages under control and LPS-treated conditions, supplemented with 10 ng bulk digests to generate a spectral library. In all projects, the larger-input runs (5–10 ng) were used to improve protein identification for the low-input (pg-level and single-cell) datasets.

Downstream data analysis and visualization were performed using in-house Python scripts. Protein-level data were filtered to exclude entries with more than 33% missing values, and intensity values were median-normalized within each group.

## Supplementary Material

Supplementary Files

This is a list of supplementary files associated with this preprint. Click to download.

- SupplementaryInformationsubmit.docx
- AccesstoMSrawdata.docx
- Supplementaryvideo1.mp4
- Supplementaryvideo2.mp4
- Supplementaryvideo3.mp4

## Figures and Tables

**Table 1:** Timeline for the 5-min multicolumn nanoLC–MS platform

1^st^ 5 min	2^nd^ 5 min	3^rd^ 5min	4^nd^ 5 min
Sample pickup	Sample pickup	Sample pickup	Sample pickup
Loading to Trap	Loading to Trap	Loading to Trap	Loading to Trap
Gradient elution from Trap #1 to storage loop #1	Gradient elution from Trap #2 to storage loop #2	Gradient elution from Trap #1 to storage loop #1	Gradient elution from Trap #2 to storage loop #2
Pushing from storage #2 to column #2	Pushing from storage #1 to column #1	Pushing from storage #2 to column #2	Pushing from storage #1 to column #1
Pre-blank 1	Pre-blank 2	1^st^ chromatogram	2^nd^ chromatogram

## Data Availability

The mass spectrometry proteomics data have been deposited to the ProteomeXchange Consortium via the PRIDE^78^ partner repository with the dataset identifier PXD070201.
